# Warming and Ventilation

**Published:** 1869-10

**Authors:** 


### WARMING AND VENTILATION.

According to the present state of human knowledge, there is only one way to have a room filled, in winter time, with warm, pure air, and that is to burn fuel liberally and keep the doors open. If but little fuel is burned, and we have double windows and miles of weather strips, the house will be warmed, but with a stenchy atmosphere, and there is no help for it.

Every year stoves and furnaces and other heating appliances are thrown upon the market which are to give more heat at a less consumption of fuel than any previous device; and taking the word of successive inventors for it, we ought, by this time, have all the heat wanted without burning any fuel at all.

Within a year an apparatus was exhibited in Broadway which promised that a tallow-candle or a gas-jet applied to a quart of water would keep a large room warm for a hundred years — a new candle, but the same water — and it was announced in the papers that books were opened for a limited number of shares of stock. But, somehow or other, the candle went out, the water evaporated, and so did the company, for summer came on and everybody had more heat than they wanted, and were willing to pay a premium to get rid of it. There has been a great outcry, too, against hot-air furnaces, which were charged with all the diseases known to man, by those who had a million miles of water and steam-piping to sell.

A good hickory-wood fire, in an old-fashioned fire-place, is just the thing to keep a room warmed with pure air. But as hickory, or any other kind of wood, is too expensive, the next best thing is to burn coal in an old-fashioned fire-place with a big throat and wide-glaring jams. This is considered the greatest domestic blessing of modern times in connection with health for house-warming, in the shape of the low-down grate, made by Dixon & Sons of Philadelphia; the heat is just as pure and cheap, and cheaper, than the old hickory fire of our grandfathers, and more cleanly and less troublesome by a very great deal.

Much has been written against hot-air furnaces, about the red-hot iron burning up all the oxygen of the air and giving a close and oppressive breathing. Then, again, certain poisonous gases were alleged to permeate the pores of the unfortunate furnace, casting death and destruction in every direction.

The next dodge, or guess, or conjecture was, that if the air was warmed, its moisture was dried out of it, and that to breathe dry air was murder. To remedy this a great, big pan of water was placed on the stove or mantle, soon simmering and filling with dust and organic matters, of various and yet no very cleanly origin, and there that dirty water steamed, and simmered, and fizzled by the hour, sending its pernicious fumes all abroad.

Then there was a theory that a lump of coal as big as your head had more heat in it, in proportion, than an egg-sized lump; hence the furnace was filled with immense boulders of coal which required half a day for ignition, then burned most furiously, scorching everybody and everything, thus freezing the family all morning and melting them at night.

It was next agreed that the receptacle for coal should be enlarged; that if a peck of coal gave out a heap of heat, a receptacle which held a bushel or two would give out heaper, and save the trouble of frequent replenishment. Still, furnaces do not adequately heat large houses in New York, and almost all in good circumstances have fires in their grates in cold weather, in addition to the furnace in the cellar, which is relied upon mainly to warm the halls only.

The gentlemen already named have contrived a furnace which obviates many, if not all of the objections just referred to, by simply having a very broad and very shallow fire-pot or coal receptacle. It is four or five inches deep, instead of a foot or more, and near two feet across, instead of half that amount. With such an arrangement, and fed every hour or two during the day with two or three shovelfulls of coal, averaging the size of a large hen's egg, several very important practical results would follow: the large surface of burning coal would give out a correspondingly larger amount of heat. That heat, regularly fed, would be diffused regularly and equably; not at any time fierce enough to burn up the iron, but always sufficient. The fresh coal thrown in would kindle within five or ten minutes, and begin to give out its own additional heat; whereas, if the coal is large, an hour would be lost in its kindling. Housekeepers have frequently noticed the irregular heat of their furnaces, and feel a great impatience at the slow warming of apartments. All these inconveniences and discomforts arise from deep and narrow fire-pots and too large-sized coal. Another advantage of the furnace spoken of is the very great simplicity of its construction, easily emptied of its ashes and its clinkers, by the withdrawal of a rod, and so little liable to get out of repair, costing half what many other complicated articles do, and really consuming less coal, and getting a very much larger amount of heat from it; and yet the smell of a "burning" stove or iron is never observed.

